# Detecting Genetic Associations between *ATG5* and Lupus Nephritis by *trans*-eQTL

**DOI:** 10.1155/2015/153132

**Published:** 2015-10-05

**Authors:** Yue-miao Zhang, Fa-juan Cheng, Xu-jie Zhou, Yuan-yuan Qi, Ping Hou, Ming-hui Zhao, Hong Zhang

**Affiliations:** ^1^Renal Division, Peking University First Hospital, Peking University Institute of Nephrology, Key Laboratory of Renal Disease, Ministry of Health of China and Key Laboratory of Chronic Kidney Disease Prevention and Treatment, Peking University, Ministry of Education, Beijing 100034, China; ^2^Shandong Provincial Hospital Affiliated to Shandong University, Jinan, Shandong 250021, China

## Abstract

*Objectives*. Numerous loci were identified to perturb gene expression in *trans*. As elevated *ATG5* expression was observed in systemic lupus erythematosus (SLE), the study was conducted to analyze the genome-wide genetic regulatory mechanisms associated with *ATG5* expression in a Chinese population with lupus nephritis (LN). *Methods*. The online expression quantitative trait loci database was searched for *trans*-expression single nucleotide polymorphisms (*trans*-eSNPs) of *ATG5*. Tagging *trans*-eSNPs were genotyped by a custom-made genotyping chip in 280 patients and 199 controls. For positive findings, clinical information and bioinformation analyses were performed. *Results*. Four *trans*-eSNPs were observed to be associated with susceptibility to LN (*P* < 0.05), including ANKRD50 rs17008504, AGA rs2271100, PAK7 rs6056923, and TET2 rs1391441, while seven other *trans*-eSNPs showed marginal significant associations (0.05 < *P* < 0.1). Correlations between the *trans*-eSNPs and *ATG5* expression and different expression levels of *ATG5* in SLE patients and controls were validated, and their regulatory effects were annotated. However, no significant associations were observed between different genotypes of *trans*-eSNPs and severity or outcome of the patients. *Conclusion*. Using the new systemic genetics approach, we identified 10 loci associated with susceptibility to LN potentially, which may be complementary to future pathway based genetic studies.

## 1. Introduction

A strong body of evidence has suggested the complex genetic basis of systemic lupus erythematosus (SLE), and to date more than 50 loci have been identified, largely improving our insights into the pathogenesis of SLE [1, 2]. However, approximately 80%–90% of the associated variants were observed to be located in noncoding regions, which may have effects on gene expressions [3, 4]. As the gene expression level has been suggested to be heritable, the expression quantitative trait loci (eQTL) have been widely studied. Up to date, a number of eQTL were observed to influence gene expression through* cis*-acting regulatory effects (with the variants located within or near the target gene) [5], significantly broadening our understanding of genetic pathogenesis of diseases [6, 7]. However,* trans*-acting regulatory effects (with the variants distal to the target gene or on different chromosomes) have been seldom addressed.

Autophagy is a phylogenetically ancient mechanism by which the cell can degrade and dispose of intracellular constituents or intracellular infectious agents in a regulated manner. Recently, genetic variants within or near* ATG5*—a gene product required for the formation of autophagosomes—have been identified to be associated with SLE by several genome-wide association studies (GWASs) [8, 9]. And elevated* ATG5* expression level was observed in the splenic and renal macrophages of lupus mice and in peripheral blood mononuclear cells (PBMC) of SLE patients [10]. However, no significant associations between variants within* ATG5* and SLE were observed in a Chinese population [9]. Also, in our previous study [11], only variants in the* PRDM1-ATG5* intergenic region not within* ATG5* were detected to be associated with susceptibility to SLE, and they were detected to affect* ATG5* expression level through a* cis-*eQTL effect. Animal studies suggested that* ATG5*-null mice were lethal within 24 hours of birth, indicating the important role of* ATG5* for life. Thus,* ATG5* was likely to be a strong susceptibility gene to SLE and its abnormal expression may be a key determinant in susceptibility. As a strong body of evidence supported that numerous loci perturb gene expression in* trans* [12–14], we thus hypothesized that genetic polymorphisms of autophagy genes may also function at the upstream of* ATG5* by* trans*-eQTL effects to further modulate SLE susceptibility.

Lupus nephritis (LN), a major phenotype with poor prognosis of SLE, is possibly a kind of extreme phenotype. It was reported that autophagy inhibitions could decrease proteinuria levels, robustly reduced renal immune complex deposition, and remitted glomerulonephritis. Thus, by choosing* trans*-expression single nucleotide polymorphisms (*trans*-eSNPs) of* ATG5* at the genome-wide level, the present genetic association study was conducted to search for the regulatory mechanisms associated with the gene expression of* ATG5* in Chinese patients with LN.

## 2. Subjects and Methods

### 2.1. Subjects

A total of 280 LN patients from the Peking University First Hospital, who were of Han ethnicity living in north of China, were enrolled in the study. Their mean age was 33.2 ± 9.8 years and 247 of them were female. The controls were 198 geographically and ethnically matched healthy blood donors. Their mean age was 34.5 ± 10.3 years and 97 of them were female. One HapMap Han Chinese in Beijing (CHB) sample (NA18524) was included as a positive control for checking genotyping and no template control was taken as a negative control. All the patients met the revised SLE criteria of the American College of Rheumatology (ACR) [15] and were confirmed by renal biopsy using light microscopy, immunofluorescence, and electron microscopy.

The study was approved by the medical ethics committee of Peking University First Hospital and all the subjects gave written informed consents.

### 2.2. SNP Selection and Genotyping

The online eQTL database (mRNA by SNP Browser v. 1.0.1, http://www.sph.umich.edu/csg/liang/imputation/byGene.html) based on Epstein-Barr virus-transformed lymphoblastoid cell lines from 400 children was used to search for* trans*-eSNPs of* ATG5.* The inclusion criteria of the* trans*-eSNPs included (1) locating within the respective genes, (2) minor allele frequency (MAF) more than 5%, and (3) call rate more than 95%. In total, 1097* trans*-eSNPs of* ATG5* were derived from the database and with the inclusion criteria 78 tagging* trans*-eSNPs (correlation coefficients between genotypes and* ATG5* expression ranged from 0.302 to 0.999,and* P* values ranged from 1.00 × 10^−6^ to 2.00 × 10^−4^) were selected for genotyping.

The tagging* trans*-eSNPs were customized into a genotyping chip by an Illumina Solexa HiSeq 2000 platform (VC-201-0144). Beadpress Scanner and illumina Genomstudio were used for the analysis of chip data.

### 2.3. Statistical Analyses

**Figure 1:**
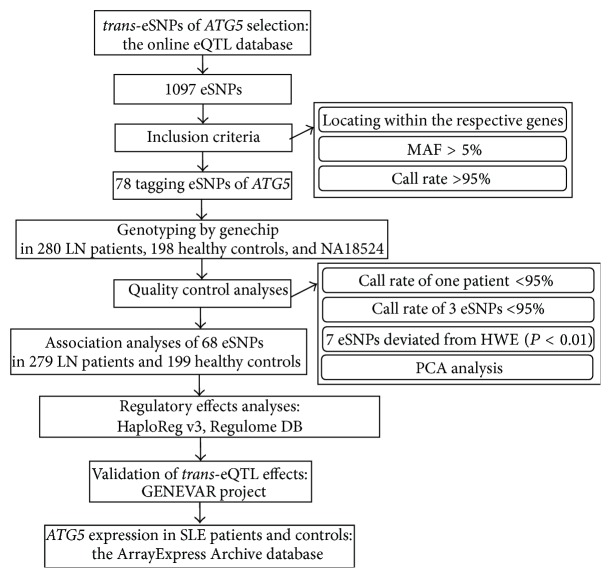
The workflow of genetic association study of* ATG5* by* trans*-eQTL. (a) eQTL: expression quantitative trait loci, eSNP: expression single nucleotide polymorphism, HWE: Hardy-Weinberg equilibrium, LN: lupus nephritis, MAF: minor allele frequency, PCA: principle component analysis, and SLE: systemic lupus erythematosus. (b) The online eQTL database: mRNA by SNP Browser v 1.0.1, http://www.sph.umich.edu/csg/liang/imputation/byGene.html. (c) HaploReg v3 database: http://www.broadinstitute.org/mammals/haploreg/haploreg_v3.php. RegulomeDB database: http://regulome.stanford.edu/. (d) GENEVAR project: the Gene Expression Variation project, http://www.sanger.ac.uk/humgen/genevar/. (e) The ArrayExpress Archive database: http://www.ebi.ac.uk/arrayexpress/.

For quality control analyses,* trans*-eSNPs were excluded if they had a call rate lower than 95% or a significant deviation from Hardy-Weinberg equilibrium in controls (*P *< 0.01). Similarly, we removed all the samples with a genotyping rate lower than 95% from further analysis. Principle component analysis (PCA) was used to detect population outliers in both cases and controls as previously described [16]. After quality control analyses for* trans*-eSNPs and samples, 68* trans*-eSNPs in 279 LN patients and 199 controls were left for further genetic association analyses (Figure 1).

Allele frequencies were compared between cases and controls using chi-square tests and Fisher's exact test was used when necessary. For the positive* trans*-eSNPs, we evaluated the associations between their genotypes and disease severity and outcome. Results of the measurement data were expressed as mean ± SD, and* t*-tests or one-way analysis of variance were used to analyze the difference. Statistical analyses were performed with SPSS16.0 software (SPSS Inc., Chicago, IL). A two-tailed* P* value of less than 0.05 was considered statistically significant.

### 2.4. Bioinformation Analyses

For noncoding variants, RegulomeDB and HaploRegv3 databases were searched for their regulatory effects. For exon variants, PolyPhen-2 (Polymorphism Phenotyping v2, http://genetics.bwh.harvard.edu/pph2/) was used to predict possible impact of an amino acid substitution on the structure and function of the protein.

To validate the* trans*-eQTL effects of the positive* trans*-eSNPs, the gene expression profiling of EBV-transformed lymphoblastoid cell lines of 270 unrelated HapMap individuals from the Gene Expression Variation project (GENEVAR project, http://www.sanger.ac.uk/humgen/genevar/) was used. Besides, the different expression levels of* ATG5* were checked between SLE (E-GEOD-50772) and LN (E-GEOD-32592 and E-GEOD-32591) and healthy controls by using the ArrayExpress Archive database (http://www.ebi.ac.uk/arrayexpress/).

## 3. Results

### 3.1. Association Analyses of* trans*-eSNPs with LN

After quality control analyses, a total of 68 tagging* trans*-eSNPs were analyzed in 279 LN patients and 199 healthy controls. Four of them, including rs17008504 on* ANKRD50* (OR = 0.645, 95% CI = 0.476 to 0.875,* P* = 0.005), rs2271100 on* AGA* (OR = 1.630, 95% CI = 1.105 to 2.405,* P* = 0.014), rs6056923 on* PAK7* (OR = 0.546, 95% CI = 0.326 to 0.916,* P* = 0.022), and rs1391441 on* TET2* (OR = 1.345, 95% CI = 1.039 to 1.743,* P* = 0.025), were observed to be associated with LN in the current study (Table 1). And, marginal significance was observed between 7/68* trans*-eSNPs and susceptibility to LN (0.05 <* P* < 0.1), including rs712377 on* SLC25A21* (OR = 1.283, 95% CI = 0.984 to 1.671,* P* = 0.065), rs1391438 on* TET2* (OR = 1.266, 95% CI = 0.978 to 1.638,* P* = 0.073), rs10878953 on* CPSF6* (OR = 1.268, 95% CI = 0.977 to 1.647,* P* = 0.075), rs7529592 on* AKNAD1* (OR = 0.701, 95% CI = 0.463 to 1.062,* P* = 0.094), rs155098 on* ITGA4* (OR = 1.264, 95% CI = 0.960 to 1.665,* P* = 0.095), rs7751485 on* CDKAL1* (OR = 1.286, 95% CI = 0.975 to 1.698,* P* = 0.096), and rs7081173 on* LINP* (OR = 1.245, 95% CI = 0.962 to 1.611,* P* = 0.096). Thus, a total of 10 loci were suggested to play a potential role in the pathogenesis of LN through* trans*-eQTL effects.

To increase the detecting power, the genotype data of 136 CHB controls (except for the positive control NA18524) from the HapMap3 project, which were available for rs2271100, rs7529592, rs7081173, rs712377, rs1391438, and rs1391441, were downloaded. Using these data and our genotyping data, association analyses were performed. As can be seen in Table 1, almost all the loci showed more significant associations with combined data.

Besides, except for analyzing the association of the SNPs with LN susceptibility, we detected the association between* trans*-eSNPs genotypes and severity and outcome of LN patients in further, including their onset age, proteinuria, estimated glomerular filtration rate (eGFR), serum creatinine level, C3 level, systemic lupus nephritis disease activity index (SLEDAI) scores, percentage of crescent, different histological classes, response to treatment, and development of end stage renal disease (ESRD). Among them, response to treatment was measured by changes in proteinuria, and complete remission was defined as proteinuria <0.3 g per 24 hours while partial remission was defined as a decrease in proteinuria by at least 50% from the initial value and <3.5 g per 24 hours. Development of ESRD was defined as dialysis or death. However, no significant differences were observed between these clinical features and the different genotypes of the positive* trans*-eSNPs of* ATG5* (see supplementary Table 1 in the Supplementary Material available online at http://dx.doi.org/10.1155/2015/153132), which may be due to the lower detecting power for subset analysis.

### 3.2. Functional Annotations by ENCODE Databases

RegulomeDB and HaploReg databases were used to annotate the regulatory effects of the positive* trans*-eSNPs. In RegulomeDB database, the* trans*-eSNPs with scores between 1 and 4 were listed (Supplementary Table 2); among them rs2271100, rs7081173, rs10788613, and rs11177577 showed the highest score (1f, eQTL + TF binding/DNase peak) (Supplementary Table 3). And in HaploReg v3 database, the positive* trans*-eSNPs were annotated to locate in the regions of promoter histone marks, enhancer histone marks, DNase-I hypersensitivity, protein binding, eQTL tissues, and regulatory motif, suggesting their potential roles for gene expression regulation (Supplementary Table 4). Besides, 1 missense variant rs10788611 (in strong linkage disequilibrium (LD) with rs7081173,* r* ^2^ = 0.98) on* LIPN* and 1 synonymous variant rs2305641 (in strong LD with rs10878953,* r* ^2^ = 0.98) on* CPSF6* were searched. However, their effects on protein function still need to be studied further.

### 3.3. Validation of* trans*-eQTL Effects of the Positive* trans*-eSNPs and the Differential Expression Level of* ATG5* in SLE

The 11 positive* trans*-eSNPs were significantly associated with the expression level of* ATG5* in our selective database of lymphoblastoid cell lines from 400 children (correlation coefficient ranged from 0.666 to 0.999, and* P* ranged from 0.00015 to 1.00 × 10^−6^). Their* trans*-eQTL effects were validated in 270 unrelated HapMap individuals. As shown in Table 2, except for rs155098 and rs7751485, the significant associations between the positive* trans*-eSNPs and* ATG5* expression level were consistently verified.

Furthermore, we ascertained whether* ATG5* was expressed differently in SLE patients and healthy controls. The expression level of* ATG5* was significantly higher in SLE PBMC and LN tubulointerstitial samples than those of controls (1794.00 ± 240.22 versus 1541.73 ± 201.64,* P* = 6.27 × 10^−5^; 6.75 ± 0.19 versus 6.46 ± 0.20,* P* = 1.82 × 10^−5^) while in glomeruli samples of LN only marginal significant association was observed (7.86 ± 0.22 versus 7.73 ± 0.15,* P* = 0.053).

## 4. Discussion

Hypothesis-free GWASs have significantly broadened our views about genetic pathogenesis of SLE [1]. However, the majority of the associated variants were noncoding variants and with modest effects (OR = 1.1–1.5), which can only account for a small proportion of heritability of SLE. Gene expression level was suggested to be heritable, and the detection of the related eSNPs was considered to be an efficient way to reconstruct gene networks [17]. As a disease-predisposing gene, higher* ATG5* expression level [10, 11] and variants with* cis*-eQTL effects within or near* ATG5* [11] were observed in SLE. Thus, we suspect that any variants that perturb the expression of* ATG5* in* trans* would also be related to the susceptibility to SLE. Thus, the present study was conducted to analyze the genome-wide genetic regulatory mechanisms associated with the gene expression of* ATG5* in Chinese LN patients. By searching the online eQTL database, 78 tagging* trans*-eSNPs of* ATG5* were genotyped for the association study. The results showed that 4* trans*-eSNPs, including rs17008504, rs2271100, rs6056923, and rs1391441, showed significant associations with susceptibility to LN (*P* < 0.05), while 7* trans*-eSNPs, including rs712377, rs1391438, rs10878953, rs7529592, rs155098, rs7751485, and rs7081173, showed marginal significant associations (0.05 <* P* < 0.1). However, no significant associations between the SNPs and severity or outcome of patients with LN were observed in the current study.* In silicon* analysis suggested their regulatory effects. Besides, compared with healthy controls, higher expression level of* ATG5* was observed in PBMC, tubulointerstitial, and glomeruli samples of SLE patients, and the correlation between the positive* trans*-eSNPs and* ATG5* expression level was validated in 270 unrelated HapMap individuals, suggesting the potential role of these loci in the pathogenesis of LN through perturbing the expression of* ATG5*.

Since* ATG5* was reported to be expressed in B cells, the eQTL database of lymphoblastoid cell lines from 400 children was chosen for searching* trans*-eSNPs of* ATG5*, for its relatively larger sample size and that environmental factors tend to have less effects on children. However, though the correlation between the* trans*-eSNPs and* ATG5* expression was validated in EBV-transformed lymphoblastoid B cell lines from the 270 unrelated HapMap individuals (JPT, CHB, CEU, and YRI), the association significance seemed to be weaker. This may be due to the smaller sample size, and also it was presumed that* trans*-eQTL effects are often more indirect and therefore weaker [14], indicating the necessity to replicate the associations in a larger cohort in the future.

In the present study, 4 loci, including* ANKRD50*,* AGA*,* PAK7*, and* TET2*, were identified to be associated with susceptibility to LN significantly. ANKRD50 (ankyrin repeat domain 50) was reported to have an essential role in the SNX27-retromer-mediated endosome-to-plasma-membrane recycling [18], but its exact mechanism was still needed to be studied further. AGA (aspartylglucosaminidase) is a lysosomal hydrolase that participates in the degradation of glycoproteins. Like other lysosomal enzymes, the deficiency of AGA leads to lysosomal storage disorder and* AGA* mutation was suggested to be weakly associated with chronic arthritis [19]. Thus, whether the dysfunction of AGA is associated with susceptibility to SLE by affecting autophagy—a degradation process conducted by lysosome—should be studied. PAK7 (p21 protein-activated kinase 7) is a member of Ser/Thr protein kinases, which has effects on cytoskeletal dynamics, cell proliferation, and survival [20]. For both ATG5 and microtubule associated light chain 3 (LC3) plays important roles in the extension of autophagosome membrane, PAK7 may regulate the expression of* ATG5* through microtubule system. TET2 (tet methylcytosine dioxygenase) is a methylcytosine dioxygenase that catalyzes the conversion of methylcytosine to 5-hydroxymethylcytosine. It was involved in chromatin modifications and other cellular processes through the interaction with O-linked *β*-N-acetylglucosamine transferase and it was also reported to be associated with several myeloproliferative disorders [21, 22]. Future studies are needed to uncover the mechanism of epigenetic and signaling networks wired with TET2 in SLE. Besides, another 6 loci with marginal associations, including* SLC25A21*,* CPSF6*,* AKNAD1*,* ITGA4*,* CDKAL1*, and* LINP*, were identified. SLC25A21 (solute carrier family 25 member 21) is a homolog of the* S. cerevisiae* ODC proteins. It is reported that HIV-1 Vif downregulates the expression of* SLC25A21* in Vif-expression T cells [23]. And, virus infection was closely associated with SLE, indicating the potential role of* SLC25A21* in SLE. CPSF6 (cleavage and polyadenylation specific factor 6) is one subunit of a cleavage factor required for 3′ RNA cleavage and polyadenylation processing. The interaction between CPSF6 and RNA is involved in the assembly of the 3′ end processing complex and facilitates the recruitment of other processing factors. AKNAD1 (AKNA domain containing 1) contains a domain found in an AT-hook-containing transcription factor and its alternative splicing can result in multiple transcript variants. ITGA4 (alpha 4 subunit of VLA-4 receptor) belongs to the integrin alpha chain family proteins, which can mediate adhesion of cells [24] and participate in B cell apoptosis. Besides, gene expression of integrins and their ligands was found to be upregulated in rheumatology arthritis [25].* CDKAL1* (CDK5 regulatory subunit associated protein 1-like 1) is a member of the methylthiotransferase family and GWASs have linked intronic SNPs of* CDKAL1* with susceptibility to type 2 diabetes [26]. As autophagy is also strongly suggested to be associated with the pathogenesis of metabolic diseases including diabetes, the interaction between* CDKAL1 *and* ATG5 *could exist. LIPN (lipase, family member N) is a lipase that is highly expressed in granular keratinocytes in the epidermis and plays a role in the differentiation of keratinocytes. Mutations in this gene were reported to be associated with lamellar ichthyosis type 4 [27]. In the present study, a missense variant rs10788611 within* LIPN* showed potential association with LN, which has caused the amino acid change from Threonine (T) to Asparagine (N) at position 244. However, its effects on protein function were seldom studied. Overall, the data above provided some clues for understanding the potential role of* ATG5* in the genetic pathogenesis of SLE.

However, there were still some limits in the present study. Due to the low allelic frequency of some* tran*-eSNPs and the relatively small sample size, the associations between* tran*-eSNPs and susceptibility to LN seemed to be weak, which could be enhanced by adding the referred CHB controls in HapMap. Besides, the smaller sample size of genotype subsets could be difficult to provide enough power for analyzing the association between* tran*-eSNPs genotypes and clinical features. Thus, to replicate the associations in a larger population is still needed in the future.

In summary, the present study established a relationship between* trans*-eSNPs of* ATG5* and LN in a northern Han population from China. By this new systemic genetics approach, 10 loci have been identified to be associated with LN potentially, widely broadening our understanding of the genetics role of* ATG5* in LN. Although these variants showed moderate associations, our approach allows for analysis of association data from a new perspective, and the results may be complementary to future pathway based genetic studies in SLE.

## Supplementary Material

The supplementary materials have provide information about the association analyses between the positive trans-eSNPs genotypes and severity and outcome of LN patients and the bioinformation analyses of these *trans*-eSNPs in RegulomeDB and HaploReg v3 databases. As shown in Supplementary Table 1, no significant differences were observed between the clinical features and the different genotypes of the positive *trans*-eSNPs of *ATG5*. In Supplementary Table 2, the *trans*-SNPs with scores between 1 and 4 were listed and Supplementary Table 3 showed the scoring standard in RegulomeDB database. And searching by HaploReg v3, the regulatory effects of the positive *trans*-eSNPs and the SNPs in strong linkage disequilibrium (r^2^ ≥ 0.8) with them were shown in Supplementary Table 4.

## Figures and Tables

**Table 1:** Allelic association analyses of the positive *trans*-eSNPs of *ATG5* in LN.

Chr	Position	Locus	SNP (minor allele)	MAF Case/ control (%)	Allele OR by minor allele (95% CI)	Allele *P* values	
						Current population	Combined with HapMap CHB population
4	124718662	5′ of ANKRD50	rs17008504(A)	19.2/26.9	0.645 (0.476, 0.875)	0.005	—
4	177438525	AGA	rs2271100(G)	16.1/10.6	1.630 (1.105, 2.405)	0.014	**0.012**
20	9840271	5′ of PAK7	rs6056923(G)	4.9/8.5	0.546 (0.326, 0.916)	0.022	—
4	105207603	TET2	rs1391441(G)	49.8/42.3	1.345 (1.039, 1.743)	0.025	**0.002**
14	36867767	SLC25A21	rs712377(C)	42.3/36.4	1.283 (0.984, 1.671)	0.065	0.146
4	105230686	TET2	rs1391438(G)	51.6/45.7	1.266 (0.978, 1.638)	0.073	**0.009**
8	69280407	3′ of CPSF6	rs10878953(G)	45.0/39.2	1.268 (0.977, 1.647)	0.075	—
1	108823932	AKNAD1	rs7529592(G)	9.0/12.3	0.701 (0.463, 1.062)	0.094	**0.080**
2	181482227	ITGA4	rs155098(G)	35.3/30.2	1.264 (0.960, 1.665)	0.095	—
6	21127081	CDKAL1	rs7751485(G)	34.8/29.3	1.286 (0.975, 1.698)	0.096	—
10	88787819	3′ of LIPN	rs7081173(A)	50.2/44.7	1.245 (0.962, 1.611)	0.096	0.116

(a) CHB: Han Chinese in Beijing, 95% CI: 95% confidence interval, chr: chromosome, LN: lupus nephritis, OR: odds ratio, SNP: single nucleotide polymorphism.

(b) *P* values were calculated by chi-square test using 2 × 2 contingency tables based on allele frequencies.

(c) None of the genotypes in the controls or patients showed significant deviation from Hardy-Weinberg equilibrium.

(d) Chromosome positions were referred to GRCh38.

**Table 2:** Correlation between genotypes of the positive *trans*-eSNP with *ATG5 *expression in public databases.

Gene	Children LCL (*n* = 400)		HapMap LCL (*n* = 270)		
	Positive *trans*-eSNPs	*P*	SNP^d^	*r* ^2^	*P*
ANKRD50	rs17008504	9.10 × 10^−5^	rs1027497	0.92	0.001
AGA	rs2271100	1.20 × 10^−4^	rs2271100	1	0.002
PAK7	rs6056923	1.10 × 10^−4^	rs6056922	1	0.001
TET2	rs1391441	5.90 × 10^−6^	rs1391441	1	0.005
TET2	rs1391438	7.10 × 10^−6^	rs7655890	0.99	0.003
SLC25A21	rs712377	6.70 × 10^−5^	rs712377	1	0.037
CPSF6	rs10878953	6.40 × 10^−5^	rs7308481	0.99	0.030
AKNAD1	rs7529592	6.00 × 10^−5^	rs7529592	1	0.001
ITGA4	rs155098	1.20 × 10^−4^	rs155099	1	0.136
CDKAL1	rs7751485	1.00 × 10^−6^	rs10946430	0.97	0.215
LIPN	rs7081173	1.50 × 10^−4^	rs7081173	1	0.054

(a) LCL: lymphoblastoid cell lines, *trans*-eSNP: *trans*-expression single nucleotide polymorphism.

(b) Children LCL refers to lymphoblastoid cell lines from 400 children from families recruited through a proband with asthma.

(c) HapMap LCL refers to Epstein-Barr virus-transformed lymphoblastoid cell lines from 270 HapMap CEU, CHB, JPT, and YRI individuals (http://www.sanger.ac.uk/humgen/genevar/).

(d) Only the SNP on each locus with strongest associated significance with *ATG5* expression level was shown.

## References

[1] Alarcón-Segovia D., Alarcón-Riquelme M. E., Cardiel M. H. (2005). Familial aggregation of systemic lupus erythematosus, rheumatoid arthritis, and other autoimmune diseases in 1,177 lupus patients from the GLADEL cohort. *Arthritis & Rheumatism*.

[2] Rullo O. J., Tsao B. P. (2013). Recent insights into the genetic basis of systemic lupus erythematosus. *Annals of the Rheumatic Diseases*.

[3] Vidal M., Cusick M. E., Barabási A.-L. (2011). Interactome networks and human disease. *Cell*.

[4] Sanyal A., Lajoie B. R., Jain G., Dekker J. (2012). The long-range interaction landscape of gene promoters. *Nature*.

[5] Stranger B. E., Nica A. C., Forrest M. S. (2007). Population genomics of human gene expression. *Nature Genetics*.

[6] Dixon A. L., Liang L., Moffatt M. F. (2007). A genome-wide association study of global gene expression. *Nature Genetics*.

[7] Cookson W., Liang L., Abecasis G., Moffatt M., Lathrop M. (2009). Mapping complex disease traits with global gene expression. *Nature Reviews Genetics*.

[8] Harley J. B., Alarcón-Riquelme M. E., Criswell L. A. (2008). Genome-wide association scan in women with systemic lupus erythematosus identifies susceptibility variants in ITGAM, PXK, KIAA1542 and other loci. *Nature Genetics*.

[9] Han J. W., Zheng H. F., Cui Y. (2009). Genome-wide association study in a Chinese Han population identifies nine new susceptibility loci for systemic lupus erythematosus. *Nature Genetics*.

[10] Li B., Yue Y., Dong C., Shi Y., Xiong S. (2014). Blockade of macrophage autophagy ameliorates activated lymphocytes-derived DNA induced murine lupus possibly via inhibition of proinflammatory cytokine production. *Clinical and Experimental Rheumatology*.

[11] Zhou X.-J., Lu X.-L., Lv J.-C. (2011). Genetic association of PRDM1-ATG5 intergenic region and autophagy with systemic lupus erythematosus in a Chinese population. *Annals of the Rheumatic Diseases*.

[12] Yvert G., Brem R. B., Whittle J. (2003). Trans-acting regulatory variation in Saccharomyces cerevisiae and the role of transcription factors. *Nature Genetics*.

[13] Cheung V. G., Nayak R. R., Wang I. X. (2010). Polymorphic cis- and trans-regulation of human gene expression. *PLoS Biology*.

[14] Montgomery S. B., Dermitzakis E. T. (2011). From expression QTLs to personalized transcriptomics. *Nature Reviews Genetics*.

[15] Tan E. M., Cohen A. S., Fries J. F. (1982). The 1982 revised criteria for the classification of systemic lupus erythematosus. *Arthritis & Rheumatism*.

[16] Zhou X.-J., Nath S. K., Qi Y.-Y. (2014). Brief Report: identification of *MTMR3* as a novel susceptibility gene for lupus nephritis in northern Han Chinese by shared-gene analysis with IgA nephropathy. *Arthritis & Rheumatology*.

[17] Zhu J., Lum P. Y., Lamb J. (2004). An integrative genomics approach to the reconstruction of gene networks in segregating populations. *Cytogenetic and Genome Research*.

[18] McGough I. J., Steinberg F., Gallon M. (2014). Identification of molecular heterogeneity in SNX27-retromer-mediated endosome-to-plasma-membrane recycling. *Journal of Cell Science*.

[19] Arvio M., Laiho K., Kauppi M. (2002). Carriers of the aspartylglucosaminuria genetic mutation and chronic arthritis. *Annals of the Rheumatic Diseases*.

[20] Gu J., Li K., Li M. (2013). A role for p21-activated kinase 7 in the development of gastric cancer. *FEBS Journal*.

[21] Ko M., Huang Y., Jankowska A. M. (2010). Impaired hydroxylation of 5-methylcytosine in myeloid cancers with mutant TET2. *Nature*.

[22] Yamazaki J., Taby R., Vasanthakumar A. (2012). Efects of TET2 mutations on DNA methylation in chronic myelomonocytic leukemia. *Epigenetics*.

[23] Kim D. Y., Kwon E., Hartley P. D. (2013). CBF*β* stabilizes HIV Vif to counteract APOBEC3 at the expense of RUNX1 target gene expression. *Molecular Cell*.

[24] McMurray R. W. (1996). Adhesion molecules in autoimmune disease. *Seminars in Arthritis and Rheumatism*.

[25] Lowin T., Straub R. H. (2011). Integrins and their ligands in rheumatoid arthritis. *Arthritis Research and Therapy*.

[26] Ghassibe-Sabbagh M., Haber M., Salloum A. K. (2014). T2DM GWAS in the Lebanese population confirms the role of TCF7L2 and CDKAL1 in disease susceptibility. *Scientific Reports*.

[27] Israeli S., Khamaysi Z., Fuchs-Telem D. (2011). A mutation in LIPN, encoding epidermal lipase N, causes a late-onset form of autosomal-recessive congenital ichthyosis. *The American Journal of Human Genetics*.

