# Supplementary material for: Detecting Genetic Associations between ATG5 and Lupus Nephritis by trans-eQTL
Source: J Immunol Res. 2015 Oct 5;2015:153132. doi: 10.1155/2015/153132 (PMC4609853; doi:10.1155/2015/153132)
Supplement: Supplementary file 1 — The supplementary materials have provide information about the association analyses between the positive trans-eSNPs genotypes and severity and outcome of LN patients and the bioinformation analyses of these trans-eSNPs in RegulomeDB and HaploReg v3 databases. As shown in Supplementary Table 1, no significant differences were observed between the clinical features and the different genotypes of the positive trans-eSNPs of ATG5. In Supplementary Table 2, the trans-SNPs with scores between 1 and 4 were listed and Supplementary Table 3 showed the scoring standard in RegulomeDB database. And searching by HaploReg v3, the regulatory effects of the positive trans-eSNPs and the SNPs in strong linkage disequilibrium (r2 ≥ 0.8) with them were shown in Supplementary Table 4. [file 153132.f1.pdf]

Supplementary Table 1 Association analyses of the positive *trans*-eSNPs genotypes and severity and outcome of patients with LN

SNPs	Genotypes	Age onset (years)	Quantity of Urine protein (g/l)	eGFR (ml/min/1.73 m^2)	Serum creatinine (umol/l)	C3 (g/l)	SLEDAI	Percentage of crescent (%)	Patient number of histologic classes (III + IV/ others)	Patients number of complete remission /partial remission/no remission	Development rate of ESRD (%)
rs10878953	GG	30.96±12.00	4.54±3.95	85.39±42.38	133.96±19.61	0.49±0.25	19.07±5.71	21.61±26.48	40/10	0/7/34	7.69
	AG	32.01±11.18	4.70±3.51	94.12±44.78	110.34±101.64	0.49±0.25	18.85±5.90	11.53±20.39	93/40	9/20/82	37.50
	AA	30.96±9.31	5.05±4.79	93.48±39.88	124.52±135.82	0.47±0.23	18.47±8.10	14.93±22.91	53/23	2/10/55	31.58
P value		0.72	0.75	0.66	0.46	0.87	0.89	0.03	0.36	0.29	0.14
rs1391438	GG	31.58±11.59	4.81±3.61	84.50±40.69	113.05±90.64	0.51±0.22	18.68±8.58	12.19±18.48	49/16	2/13/45	29.41
	AG	31.10±10.30	5.00±4.45	94.90±44.92	127.55±142.27	0.49±0.25	18.71±5.74	14.55±23.44	92/45	5/16/89	32.35
	AA	32.30±11.18	4.19±3.18	97.00±40.45	104.64±78.08	0.45±0.23	19.08±5.47	16.99±25.08	45/12	4/8/37	23.08
P value		0.76	0.44	0.36	0.45	0.38	0.94	0.50	0.19	0.60	0.93
rs1391441	AA	32.51±11.42	4.75±3.51	84.41±41.74	114.14±93.20	0.53±0.22	19.10±8.40	11.98±18.60	45/15	2/12/41	27.78
	AG	30.69±10.27	5.11±4.51	94.32±44.86	128.59±142.01	0.47±0.25	18.54±6.02	14.96±23.44	94/44	5/16/92	33.33
	GG	32.25±11.38	4.05±3.06	97.92±39.52	101.66±75.91	0.48±0.25	18.93±5.35	15.93±24.59	47/14	4/9/38	23.08
P value		0.43	0.22	0.35	0.34	0.31	0.85	0.59	0.35	0.58	0.82
rs155098	GG	32.94±12.18	4.51±3.05	98.62±43.91	113.95±113.59	0.49±0.25	17.80±6.93	20.34±23.06	25/5	3/5/18	20.00
	AG	31.12±10.24	4.88±3.84	88.69±45.56	132.72±142.18	0.48±0.22	18.10±6.58	15.81±25.51	84/39	4/20/83	30.77
	AA	31.52±11.12	4.71±4.40	94.99±39.82	103.63±82.66	0.49±0.26	19.93±6.34	11.29±18.20	77/29	4/12/70	32.14
P value		0.70	0.88	0.57	0.19	0.97	0.12	0.12	0.26	0.42	0.87
rs17008504	AA	38.60±3.05	1.85±1.31	98.72±30.09	74.40±13.45	0.42±0.16	19.00±11.47	17.91±14.42	3/1	1/0/4	0.00
	AT	30.76±11.03	4.53±3.06	96.22±45.98	110.62±102.06	0.48±0.23	18.66±5.43	16.38±26.16	67/28	4/13/58	31.58

	TT	31.70±10.79	4.99±4.45	89.85±41.40	125.18±128.96	0.49±0.25	18.85±7.02	13.24±20.50	116/44	6/24/109	29.55
P value		0.27	0.17	0.66	0.46	0.72	0.98	0.53	0.91	0.51	1.00
	GG	28.36±12.75	4.64±4.04	100.75±50.80	125.45±94.40	0.44±0.29	19.33±5.68	9.73±10.80	10/1	0/2/7	50.00
rs2271100	AG	33.97±11.30	4.58±3.87	92.21±41.38	117.41±125.55	0.50±0.24	20.13±7.75	13.10±21.39	43/16	3/9/44	28.57
	AA	30.85±10.45	4.84±4.05	92.00±43.33	118.98±118.43	0.48±0.24	18.31±6.15	15.22±23.57	133/56	8/26/120	28.26
P value		0.08	0.91	0.87	0.98	0.74	0.21	0.64	0.39	0.98	0.71
	GG	-	-	-	-	-	-	-	-	-	-
rs6056923	AG	34.12±12.18	3.83±3.32	78.64±34.27	132.55±136.68	0.57±0.28	16.94±4.07	12.36±26.23	16/8	1/2/21	28.57
	AA	31.28±10.64	4.84±4.05	93.53±43.44	117.76±117.46	0.48±0.24	18.96±6.72	14.66±22.35	169/65	10/35/149	30.36
P value		0.20	0.25	0.27	0.60	0.13	0.23	0.64	0.64	0.61	1.00
	AA	32.66±12.12	4.82±4.39	91.39±42.79	100.08±65.75	0.50±0.27	19.28±7.71	16.59±23.78	44/21	4/10/42	16.67
rs7081173	AC	31.69±9.60	4.69±4.14	94.19±43.45	123.83±130.54	0.48±0.24	18.86±6.09	12.85±22.37	91/38	5/15/85	38.71
	CC	29.93±11.65	4.86±3.28	89.36±42.87	128.09±133.98	0.48±0.20	18.06±6.28	15.74±22.21	51/14	2/12/44	26.67
P value		0.32	0.95	0.85	0.34	0.83	0.62	0.49	0.37	0.74	0.31
	CC	30.18±10.55	4.29±3.86	101.82±47.03	95.58±60.01	0.52±0.23	20.69±5.37	15.21±24.30	29/10	1/7/26	27.27
rs712377	AC	31.57±10.54	5.23±4.42	85.21±40.39	123.18±119.80	0.48±0.25	18.27±6.96	14.78±21.56	104/36	5/19/93	29.73
	AA	32.00±11.49	4.15±3.06	99.85±43.63	122.06±136.13	0.48±0.24	18.82±6.23	13.65±23.98	53/27	5/11/52	31.25
P value		0.68	0.11	0.09	0.45	0.69	0.18	0.92	0.42	0.84	1.00
	GG	32.75±10.87	2.62±2.94	106.91±31.08	73.00±3.92	0.41±0.14	19.33±2.08	6.13±10.49	3/1	0/1/3	50.00
rs7529592	AG	30.40±10.04	4.77±3.27	96.22±45.02	142.81±177.68	0.41±0.20	19.80±7.93	11.00±21.56	30/8	1/6/27	37.50
	AA	31.67±10.98	4.81±4.13	91.50±42.98	115.89±107.39	0.50±0.25	18.61±6.38	15.25±22.97	153/64	10/30/141	27.78
P value		0.76	0.56	0.76	0.35	0.12	0.65	0.43	0.64	0.91	0.58
rs7751485	CC	34.97±10.95	4.57±3.00	91.40±41.57	89.61±53.05	0.49±0.24	20.35±4.96	10.66±15.42	17/9	2/4/18	12.50

CG	30.76±11.15	4.32±3.52	96.75±40.62	108.53±102.48	0.49±0.26	19.16±6.06	14.01±20.91	88/38	5/16/86	34.15
GG	31.47±10.29	5.36±4.65	88.05±45.83	138.98±144.12	0.49±0.22	17.96±7.37	16.00±25.92	81/26	4/17/67	26.67
P value	0.17	0.13	0.50	0.07	1.00	0.21	0.53	0.47	0.79	0.51

(a) ESRD: end stage renal disease; LN: lupus nephritis, SLEDAI: systemic lupus nephritis disease activity index;

(b) Response to treatment was measured by changes in proteinuria, and complete remission was defined as proteinuria <0.3g per 24 hours while partial remission defined as a decrease in proteinuria by at least 50% from the initial value and <3.5g per 24 hours. Development of end stage renal disease was defined as dialysis or death.

Supplementary Table 2 Function prediction of the positive *trans*-eSNPs in RegulomeDB database

Chromosome	Locus	SNP	r^2	RegulomeDB score
2	ITGA4(rs155098)	rs155099	1	3a
4	AGA(rs2271100)	rs2271100	1	1f
4	AGA	rs11726465	0.97	3a
4	TET2(rs1391441)	rs1391441	1	4
6	CDKAL1(rs7751485)	rs4479917	0.96	2b
6	CDKAL1	rs4315997	0.97	4
10	LINP(rs7081173)	rs7081173	1	1f
10	LINP	rs10788613	0.99	1f
10	LINP	rs139608926	0.95	4
12	CPSF6(10878953)	rs11177577	1	1f
12	CPSF6	rs138867102	0.98	3a
14	SLC25A21(rs712377)	rs1154121	0.9	4
14	SLC25A21	rs848045	0.82	4
20	PAK7(rs6056923)	rs36001827	1	4

(a)*trans*-eSNP: *trans*-expression single nucleotide polymorphism;

(b)Only the SNPs with RegulomeDB scores between 1 and 4 were listed.

Supplementary Table 3 The scoring standard of RegulomeDB database

Score	Supporting data
1a	eQTL + TF binding + matched TF motif + matched DNase Footprint + DNase peak
1b	eQTL + TF binding + any motif + DNase Footprint + DNase peak
1c	eQTL + TF binding + matched TF motif + DNase peak
1d	eQTL + TF binding + any motif + DNase peak
1e	eQTL + TF binding + matched TF motif
1f	eQTL + TF binding / DNase peak
2a	TF binding + matched TF motif + matched DNase Footprint + DNase peak
2b	TF binding + any motif + DNase Footprint + DNase peak
2c	TF binding + matched TF motif + DNase peak
3a	TF binding + any motif + DNase peak
3b	TF binding + matched TF motif
4	TF binding + DNase peak
5	TF binding or DNase peak
6	other

(a) eQTL: expression quantitative trait loci, TF: transcriptional factor.

Supplementary Table 4 Function prediction of the positive *trans*-eSNPs in HaploReg database v3

Gene	SNP	Function annotation[a]	Promoter histone marks[b]	Enhancer histone marks[b]	DNAse[b]	Protein bound[b]	Regulatory motifs[c]
ANKRD50	rs17008504	-	0	2	0	0	6
AGA	rs2271100	intronic	0	3	3	3	29
PAK7	rs6056923	intronic	3	2	7	0	121
TET2	rs1391441	intronic	0	6	2	0	27
TET2	rs1391438	intronic	0	6	2	0	27
SLC25A21	rs712377	intronic	0	4	2	1	63
CPSF6	rs10878953	intronic, synonymous	2	3	5	2	29
AKNAD1	rs7529592	intronic	0	0	0	0	0
ITGA4	rs155098	intronic	0	0	1	1	39
CDKAL1	rs7751485	intronic	0	4	2	1	27
LIPN	rs7081173	intronic, missense	0	3	2	1	34

(a)SNP: single nucleotide polymorphism;

(b)[a]The types of function annotation of the positive eSNPs and SNPs in strong linkage disequilibrium with them;

(c) [b]The number of cell types for validating the regions of promoter histone, enhancer histone, DNAse, or transcriptional factor binding;

(d) [c]The numbers of regulatory motifs.
